# Correction notice

**DOI:** 10.1080/10717544.2022.2127213

**Published:** 2022-09-22

**Article title:** Nano-drug co-delivery system of natural active ingredients and chemotherapy drugs for cancer treatment: a review

**Authors:** Bingqian Li, Huili Shao, Lei Gao, Huan Li, Huagang Sheng, and Liqiao Zhu

**Journal:** *Drug Delivery*

**Bibliometrics:** Volume 29, Number 1, pages 2130–2161

**DOI:** https://doi.org/10.1080/10717544.2022.2094498

As per author’s request, the captions of figure 6 and 8 should be revised as shown below.

Figure 6. Illustration of the preparation, dual-drugs loading, pH-responsive intracellular release of MSNCA@GODOX-HA nanoparticles and the apoptosis induction in MCF-7 cells. *International Journal of Nanomedicine* 2020; 15: 10285–10304’ Originally published by and used with permission from Dove Medical Press Ltd.

Figure 8. Schematic representation of the carrier-free nanoparticles (NPs) via co-assembly between UA and MTX. *International Journal of Nanomedicine* 2021; 16: 1775–1787’ Originally published by and used with permission from Dove Medical Press Ltd.

